# Serum leucine-rich alpha-2-glycoprotein-1 with fucosylated triantennary N-glycan: a novel colorectal cancer marker

**DOI:** 10.1186/s12885-018-4252-6

**Published:** 2018-04-11

**Authors:** Eiji Shinozaki, Kazuhiro Tanabe, Takashi Akiyoshi, Tomohiro Tsuchida, Yuko Miyazaki, Nozomi Kojima, Masahiro Igarashi, Masashi Ueno, Mitsukuni Suenaga, Nobuyuki Mizunuma, Kensei Yamaguchi, Konosuke Nakayama, Sadayo Iijima, Toshiharu Yamaguchi

**Affiliations:** 1 Department of Gastrointestinal Oncology, Cancer Institute Hospital of Japanese Foundation for Cancer Research, Tokyo, Japan; 2 Medical Solution Promotion Department, Medical Solution Segment, LSI Medience Corporation, 3-30-1 Shimura, Itabashi-ku, Tokyo, Japan; 3 Department of Gastrointestinal Surgery, Cancer Institute Hospital of Japanese Foundation for Cancer Research, Tokyo, Japan; 4 Department of Gastroenterology, Cancer Institute Hospital of Japanese Foundation for Cancer Research, Tokyo, Japan; 5 Biotechnology Laboratory Mitsubishi Chemical Group Science and Technology Research Center, Inc, Tokyo, Japan; 6 Department of Internal Medicine, Cancer Institute Hospital of Japanese Foundation for Cancer Research, Tokyo, Japan; 7 International Sales Department, LSI Medience Corporation, Tokyo, Japan; 8 Cancer Institute Hospital of Japanese Foundation for Cancer Research, Tokyo, Japan

**Keywords:** Leucine-rich alpha-2-glycoprotein-1, Fucosylation, N-glycan, Colorectal cancer, Tumor marker

## Abstract

**Background:**

Carcinoembryonic antigen (CEA) and carbohydrate antigen (CA)19–9 are used in clinical practice as tumor markers to diagnose or monitor colorectal cancer (CRC) patients, However, their specificities and sensitivities are not ideal, and novel alternatives are needed. In this study, mass spectrometry was used to search for screening markers, focusing on glycan alterations of glycoproteins in the sera of CRC patients.

**Methods:**

Glycopeptides were prepared from serum glycoproteins separated from blood samples of 80 CRC patients and 50 healthy volunteers, and their levels were measured by liquid chromatography time-of flight mass spectrometry (LC–TOF–MS).

**Results:**

Leucine-rich alpha-2-glycoprotein-1 with fucosylated triantennary N-glycan (LRG–FTG) was identified as CRC marker after evaluating 30,000 candidate glycopeptide peaks. The average LRG–FTG level in CRC patients (1.25 ± 0.973 U/mL) was much higher than that in healthy volunteers (0.496 ± 0.433 U/mL, *P* < 10^− 10^), and its sensitivity and specificity exceeded those of CA19–9. The combination of CEA and LRG–FTG showed a complementary effect and had better sensitivity (84%), specificity (90%), and AUC (0.91 by ROC analysis) than each marker alone or any other previously reported marker. LRG–FTG alone or combined with CEA also corresponded well with patient response to treatment.

**Conclusions:**

We identified LRG–FTG as a new CRC marker, with a sensitivity and specificity exceeding CA19–9. The combination of LRG–FTG and CEA showed much higher sensitivity and specificity than each marker alone. Further validation beyond this initial exploratory cohort is warranted.

**Electronic supplementary material:**

The online version of this article (10.1186/s12885-018-4252-6) contains supplementary material, which is available to authorized users.

## Background

The International Agency for Research on Cancer (IARC), of the World Health Organization (WHO) (http://www.irac.fr) reported that colorectal cancer (CRC) is the fourth most common cause of cancer death. Worldwide, more than 600,000 patients die of CRC every year [1]. According to the American Cancer Society (http://www.cancer.org), the 5-year overall survival of Stage I CRC patients is 90%, but is less than 20% in patients who are stage IV when diagnosed [2]. Detecting early stage cancer is crucial for saving patient lives. Endoscopy is the most reliable medical CRC screening method, but it has high economic and physical burdens. On the other hand, blood tests are affordable and easily performed, but accuracy of early-stage cancer detection is limited.

Tumor markers such as carcinoembryonic antigen (CEA) and carbohydrate antigen (CA)19–9 are used in clinical practice to diagnose and monitor CRC patients, but their specificities and sensitivities are unsatisfactory. CEA, first reported by Gold and Freedman in 1965 [3], is a member of the immunoglobulin superfamily and thought to be involved in intracellular adhesion. High serum CEA is strongly associated with malignancies, such as CRC, breast, gastric, and pancreatic cancer, and some studies have reported an association of increased pre surgery serum CEA with risk of recurrence and poor prognosis [4]. However, CEA is not specific to malignancies and is often elevated also in benign diseases, such as liver cirrhosis, gallbladder inflammation, or metabolic syndrome [5]. CA 19–9, a ligand of E-selectin that promotes binding of cancer cells to cellular endothelium, is used as a CRC or pancreatic cancer marker. Previous publications have reported cancer-related sensitivities of 50% to 90% and specificities of 54% to 98%) [6–8], but CA19–9 is also elevated in benign diseases [9], and is not detectable in the 7% of the population who are negative for the Lewis antigen. Proteomics [10], metabolomics [11], micro-RNA [12–14], and circulating cell-free DNA [15, 16] have been used to identify new highly sensitive and specific candidate CRC markers, but have not been successful.

Aberrant glycosylation of serum glycoproteins is often observed in cancer patients [17–19], particularly fucosylation following activation of fucosyltransferases [20]. Increased fucosylation of the L3 isoform of α-fetoprotein is a sensitive and specific marker of hepatocellular carcinoma [21]. Elevation of sialyl-Lewis X antigen, a tetrasaccharide carbohydrate with the sequence Neu5Acα2–3Galβ1–4[Fucα1–3]GlcNAcβ, in tri−/tetraantennary-N-linked oligosaccharides has been reported in the sera of liver and lung cancer patients [22, 23]. Although alteration of sugar chains shows promise as a cancer marker, difficulties in the analysis of the wide variety of sugar chain structures have hampered this approach.

Recent improvements of mass spectrometry in the sensitivity, resolution, and ability to rapidly analyze a large number, have revolutionized the screening of markers, specifically glycoproteins. In this study, we aimed to identify new glycoproteins as CRC markers. Our strategy was to use mass spectroscopy and liquid chromatography to analyze total glycoproteins in the sera of CRC patients and then compare candidate biomarker levels in patients with those in healthy volunteers. The advantages of this strategy are the ability to assess not only alterations of sugar chains but also those of the “host” proteins, and the ability to screen thousands (approximately 10,000 to 100,000) of glycopeptide candidates. Although screening of glycopeptide markers has been described for some specific proteins, such as haptoglobins [24, 25], screening of total serum glycoproteins has not been reported. We tried to find new CRC markers with potential as alternatives or complementarity to currently available markers.

## Methods

### Collection of blood samples and clinical data

We enrolled patients at the Cancer Institute Hospital of the Japanese Foundation for Cancer Research (Tokyo, Japan) with histologically confirmed adenocarcinoma of the colon or rectum. Patients with impaired renal and liver function were excluded. After obtaining informed consent from eligible patients, we assayed 2-mL of sera that remained after performing routine laboratory tests before surgery or chemotherapy, and at 1, 3, and 6 months after anticancer treatment. Clinical data were retrieved from patient medical records, and CT images were evaluated for antitumor effect by Response Evaluation Criteria in Solid Tumors (RECIST) version 1.1. Patient characteristics including sex, age, stage, tumor histology, and treatment were collected. We obtained sera of healthy volunteers from SOIKEN (Osaka Japan) with informed consent. All healthy volunteers received medical examinations prior to blood sampling, and those whose results exceeded normal criteria in any test were not enrolled in the study. CA19–9 and CEA were analyzed by chemiluminescent immunoassay method in the Cancer Institute Hospital. This study was approved by the Institutional Review Board (No. 2011–1025) of Cancer Institute Hospital of Japanese Foundation for Cancer Research, and was conducted following the Ethical Guidelines of Ministry of Health, Labor and Welfare in Japan. Written informed consent was obtained from all patients before participation.

### Serum preparation

Sera were prepared by previously described methods [26, 27]. Briefly, 400 μL of cold acetone containing 10% trichloroacetic acid (Wako Pure Chemical Industries, Ltd., Osaka, Japan), and 50 μg of fetal calf fetuin (Sigma, St. Louis, MO, USA) as an internal standard, were added to 100 μL of patient sera, and mixed at − 20 °C for 90 min to remove serum albumin. After the mixture was centrifuged at 13000 g at 4 °C for 20 min, the supernatants were removed and the precipitates were washed with 400 μL of cold acetone to remove excess trichloroacetic acid. After 13,000 g centrifugation, the precipitates were mixed with 1 mL denaturing solution, 40% (*w*/*v*) of urea (Wako Pure Chemical Industries), 0.5 M Tris-HCl buffer (pH 8.5), 5 mM EDTA, 40 mM Tris (2-carboxyethyl) phosphine hydrochloride (Sigma). Then 100 μL of 1 M 2-iodoacetamide (Wako Pure Chemical Industries) solution was added to denatured proteins and reacted at 37 °C for 1 h to protect the free thiol residues. The solutions were transferred into Amicon Ultra 30 K 4-mL centrifugal filtration tubes (Millipore Corp., MA, USA) and centrifuged at 3000 g for 30 min to remove denaturing reagents. The proteins trapped on the filters were washed with 2 mL of 0.1 M Tris-HCl buffer (pH 8.5), and then they were centrifuged at 3000 g for 40 min. Next, 1 mL of 0.1 M Tris-HCl buffer (pH 8.5), 100 μL of 0.1 μg/μL trypsin solution, and 100 μL of 0.1 μg/μL lysyl endopeptidase (Wako Pure Chemical Industries) solution were added into the Amicon tubes and the proteins were digested at 37 °C for 16 h. The solution was centrifuged at 3000 g for 10 min, and the supernatant containing peptides was transferred into an Amicon Ultra 10 K, 4-mL tube (Millipore Corp.), then it was centrifuged at 3000 g for 10 min. Most peptides with sugar chains were trapped on the 10 K ultra-filter, whereas most nonglycosylated peptides were removed by the filtration. The glycopeptides trapped on the filter were washed with 2 mL of a solution containing 10% acetonitrile and 90% 10 mM ammonium acetate, then they were transferred into a 1.5-mL microtube to be dried up by vacuum centrifuging.

Two mg of Aleuria aurantia lectin (AAL, Vector, Burlingame, CA, USA) hold on one mL of agarose gel was placed in an empty one-mL column (Agilent, CA, USA) and it was washed with 30 mL of 0.1 M Tris-HCl buffer (pH 7.4). Enriched glycopeptide fraction prepared from 40 μL of serum, was dissolved in 200 μL of water and loaded onto the lectin column. After it was washed with 15 mL of 10 mM Tris-HCl buffer (pH 7.4), only fucosylated glycopeptides were eluted with 15 mL of 100 mM fucose solution. The eluted solution was transferred into an Amicon Ultra 3 K tubes (15 mL) and centrifuged at 4000 g for 90 min to remove excess fucose. The fucosylated glycopeptides remaining on the filter were washed with further 10 mL of water and they were transferred into LC vial tubes to be analyzed by mass spectroscopy (LC–MS).

### Screening of CRC markers by LC–MS

LC–MS data sets were acquired with a liquid chromatograph (Agilent HP1200, Agilent Technologies, Palo Alto, CA), an electrospray ionization quadrupole and time of flight (Q-TOF) mass spectrometer (Agilent 6520, Agileent Technologies). An Inertsil C18 column (2 μm, 100 mm × 1.5 mm ID, GL Science, Tokyo, Japan) was used for high-performance liquid chromatography (HPLC). Solvent A was 0.1% formic acid aqueous solution and solvent B was 0.1% of formic acid, 9.9% water and 90% acetonitrile. Glycopeptides were eluted at a flow rate of 0.1 mL/min at 40 °C with a linear gradient of 10%–56% of Solvent B over 40 min and a further 10 min hold at 100% of Solvent B. The mass spectrometer was operated in the negative mode. The capillary voltage was set at 4000 V. Nebulizing gas pressure was 30 psi, and the dry gas flow was 8 L/min at 350 °C.

### Screening of CRC markers

The mass spectrometry data was analyzed using Marker Analysis, the software developed in our laboratory [27]. After all peak positions (retention time and m/z) and intensities (peak areas) were calculated, the peaks of all samples from patients and healthy volunteers were aligned, to generate a peak list. The errors generated in preparation and the LC–MS analysis step, were corrected using the internal standard peptides derived from fetal calf fetuin, and were normalized against glycopeptides obtained from a healthy person (HEA219). Marker screening analysis was performed by t-test statistics, mean-fold change analysis, and receiver operating characteristic (ROC) curve analysis using Marker Analysis and SPSS 17.0 (SPSS, Chicago, IL). Eighty CRC patients and 50 healthy volunteer control persons were compared. ROC curve analysis was used to determine optimum cutoff values of candidate markers and maximize sensitivity and specificity.

### Identification of CRC markers

We previously established a database of retention times and m/z of glycopeptides generated from trypsin digestion of standard proteins. Standard human serum glycoproteins, such as alpha-1-acid glycoprotein, alpha-1-antitrypsin, clusterin, haptoglobin, kininogen, leucine rich glycoprotein, alpha-2-macrogloblin, ceruloplasmin, transferrin, immunoglobulin, and complement C3, were obtained from Sigma (St. Louis, MO, USA). After these proteins were digested by trypsin, and the glycopeptides were analyzed by LC–MS, they were further treated with PNGase F (New England Biolabs, Ipswich, MA, USA) to remove N-glycans. The sugar-free peptides were analyzed by tandem mass spectroscopy (MS/MS), and the data were analyzed by Mascot (Matrix Science, version 2.3.02), using the UniProt/Swiss-Protein sequence database (October 2015, 20,266 total sequences). Database search parameters were restricted to one missed tryptic cleavage site, a precursor ion mass tolerance of 1.2 Da, a fragment ion mass tolerance of 0.6 Da and a *P*-value of < 0.05. The sugar chain compositions were suggested by the delta of molecular weight between pre- and post- PNGase F glycopeptide digestion. The analytical data (retention time and m/z of each peptide) was registered with the protein names, the sugar chain binding sites, and the proposed sugar chain structures. All glycopeptide peaks detected in CRC and healthy volunteer sera were imported into the database and assigned protein and sugar chain structures.

## Results

### Screening and identification of new CRC biomarker

**Fig. 1:**
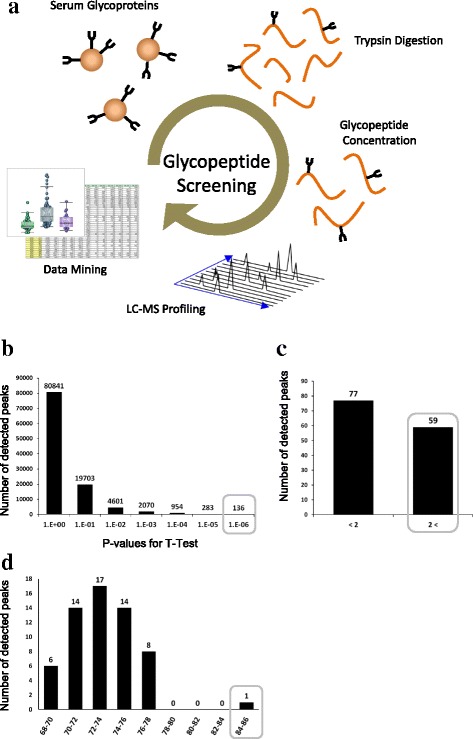
Screening of CRC cancer markers. **a** All isolated serum glycoproteins were digested by trypsin to form peptides, and the glycopeptides were enriched by ultrafiltration and AAL lectin chromatography. Then they were analyzed by LC–TOF–MS. Glycopeptide peak positions (m/z and elution time) and peak intensities (peak areas) were calculated by software developed in our laboratory. The glycopeptide peaks obtained for all serum samples were then aligned and included in a single table, i.e., a peak list. Finally, CRC markers were screened by t-test statistics, mean-fold change analysis, and ROC analysis. CRC markers were extracted with t-test values *P* < 10^− 6^, (**b**), mean-fold change analysis with ratios > 2 (**c**), and ROC analysis with AUCs > 0.80 (**d**). The values of the marker were normalized against levels of healthy controls (HEA219)

**Fig. 2:**
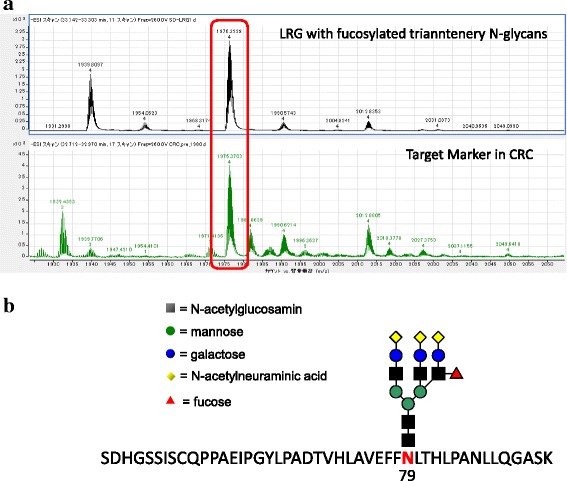
Identification of CRC marker glycopeptides. The mass spectrum of the CRC marker was incorporated into a database that included the m/z and retention times of glycopeptides generated from standard serum proteins by trypsin digestion. (**a**) mass spectrum of standard LRG glycopeptides (upper), and mass spectrum of target marker glycopeptides detected in CRC patients (lower), and (**b**) the proposed structure of the CRC marker

Eighty CRC patients and 50 healthy volunteers were enrolled (Table 1), and follow up samples were obtained from 68 of the patients. A new CRC marker was identified after screening over 30,000 glycopeptide peaks following the schema shown in Fig. 1a. First, 136 CRC peaks (0.13%) with a t-test *P* < 10^− 6^ compared with healthy volunteers (Fig. 1b) were selected. Of these, 59 peaks (0.026%) with a mean-fold change > 2 were selected (Fig. 1c), and finally one glycopeptide with an area under the curve (AUC) by ROC curve analysis > 0.80 was isolated as the best CRC marker candidate (Fig. 1d). The elution time (33 min) and m/z (1975.37, z4) of this marker was just coincident with leucine-rich alpha-2-glycoprotein-1, whose Asn79 was modified with fucosylated triantennary N-glycan (LRG–FTG, Fig. 2a, b). The sugar chain structure was proposed as A3G3S3F from the molecular weight delta of pre- and post-PNGase digestion. The sugar chain composition was proposed as six hexoses, five hexNAcs, three N-acetyl neuraminic acids, and one fucose, Fig. 2b). The average LRG–FTG concentration in CRC patients was 1.25 ± 0.973 U/mL and 0.496 ± 0.433 U/mL in healthy volunteers (P < 10^− 10^ by t-test). LRG with biantennary glycan did not change between CRC patients and healthy individuals (Additional file 1: Figure S1), which meant that the sugar chain alteration in LRG was key to cancer diagnosis.

**Table 1:** Participant characteristics

	chemotherapy	surgery	healthy individuals
sex male/female	27/28	16/9	25/25
age median(min-max)	63 (39–80)	61 (27–77)	39 (21/64)
stage I/II/III/IV	0/0/6/46	2/11/12/0	–
pathology por/mod/well^a^	3/29/19	0/15/10	–
N	55	25	50

^a^por; poorly differentiated/mod; moderately differentiated/well; well differentiated

### Comparison of LRG–FTG with current markers, CEA and CA19–9

**Fig. 3:**
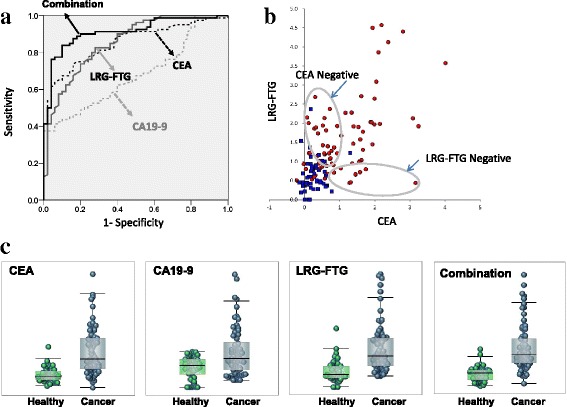
Diagnostic performance of LRG–FTG, and currently used CRC markers (CEA and CA19–9). **a** ROC curves comparing 80 CRC patients with 50 healthy volunteers for CEA, CA19–9, LRG–FTG, and the combination. **b** Box and whisker plots of CEA, CA19–9, LRG–FTG and the combination. **c** Scatter plots of CEA (Log10) and LRG–FTG. Red and blue circles represent CRC and healthy volunteers respectively. Combination values are calculated as: Combination factor = Log10 (*CEA*) × 0.8 + (*LRG–FTG*) × 0.6

Figure 3 shows box and whisker plots and ROC curves that compare the AUCs of LRG–FTG, CEA and CA19–9 in patients and healthy volunteers. The AUC of LRG–FTG (0.86) was significantly greater than that of CA19–9 (0.68), but was almost equal to that of CEA (0.85, Fig. 3a). The sensitivity of LRG–FTG was 80%, and the specificity was 74%, when the cutoff value was 0.82 U/mL. The cutoff value was the one that resulted in the highest sensitivity and specificity.

There was a weak correlation between LRG–FTG and CEA (*r* = 0.61), however their relation was complementary, as 24 of 33 (73%) CEA-negative (< 5 ng/mL) patients would be considered positive by LRG–FTG (Fig. 3b). Furthermore, a combination factor, calculated by the next formula, resulted in a much higher AUC (0.91) compared with CEA or LRG–FTG alone (Fig. 3a, c). The coefficients of this formula were optimized by EXCEL Solver program (Additional file 2).

Combination factor = Log10 (*CEA*) × 0.8 + (*LRG–FTG*) × 0.6.

The sensitivity and specificity of the combination became 84% and 90% respectively when the cutoff was 0.92 U/mL.

### Multiple glycopeptide combination screening

Orthogonal partial least squares discriminant analysis (OPLS-DA, SIMCA, Umetrics, Sweden) using 136 glycopeptides screened by t-test was performed with an aim to separate CRC patients from healthy individuals. The Q2 score, a predictive value using cross validation technique, showed reliable level (0.37) and the ROC-AUC using t1 score indicated high level (0.92, Additional file 3: Figure S2).

### Correlation of LRG–FTG and short-term treatment outcome

**Fig. 4:**
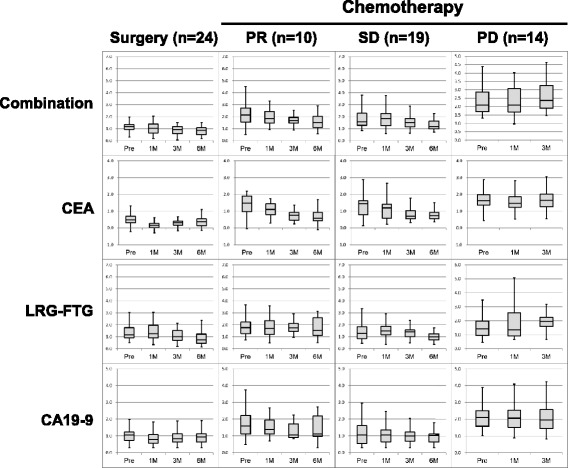
Response to chemotherapy and change in LRG–FTG, CEA, and combination values within 6 months for surgery or chemotherapy. PR, partial response; SD, stable disease; PD, progressive disease

To investigate the correlation of LRG–FTG and outcomes of surgery or chemotherapy, the 68 patients who were available for follow-up were monitored for 6 months after treatment. Prior to analysis, the patients were assigned to four groups. The first included 25 patients with complete surgical resection and no metastases or recurrences within the 6-month monitoring period. The other three groups were defined by the effectiveness of chemotherapy as partial response (PR, *n* = 10), stable disease (SD, *n* = 19), and progressive disease (PD, *n* = 14, Table 2). Changes in the LRG–FTG, CEA, and combined values observed in the four groups are shown in Fig. 4. Compared three months after treatment to pre-treatment of PD patients, LRG-FTG showed good correlation with treatment outcome (elevated in 11/14 patients), whereas CEA did not correspond well with the outcome (elevated in 6/14 patients, Additional file 4). The combined LRG–FTG and CEA value was the most closely associated with patient outcome. It continuously increased in patients with PD and decreased in those with a PR. In the surgery group, the level gradually decreased during follow up and was below the cutoff of 0.92 U/mL at 6 months.

**Table 2:** Treatment outcomes 6 months following surgery or chemotherapy

Chemotherapy	
RECIST CR/ PR/ SD/ PD ^a^	0/10/19/14
Surgery	
R0/ others	25/0
Recurrence yes/ no	0/25

^a^CR, complete response; PR, partial response; SD, stable disease; PD, progressive disease

## Discussion

In this study, we identified LRG–FTG as a new CRC marker, with a sensitivity and specificity exceeding CA19–9. Its sensitivity and specificity were almost equal to CEA, but when LRG–FTG and CEA were combined, the effect was complementary, achieving a sensitivity of 84%, specificity of 90%, and AUC of 0.91 by ROC analysis, all of which were higher than each marker alone.

LRG (leucine-rich alpha-2-glycoprotein-1) is a protein involved in an the acute-phase response to bacterial or viral infection [28], or the initiation of granulocyte differentiation. Elevation of serum LRG has been observed in various disease states, including toxic-shock syndrome [29], inflammation in cystic fibrosis [30], ovarian cancer [31], pancreatic cancer [32–34]. Wang et al. reported that LRG promoted angiogenesis in cancer progression by binding to the transforming growth factor (TGF)-β accessory receptor [35], and was considered as a potential cancer marker. In this patient series, as LRG–FTG was only weakly correlated (*r* = 0.47) with an inflammatory marker, CRP (Additional file 5: Figure S3), the relationship might be incompletely dependent.

There have been some reports of increased serum LRG in cancer patients, but few have mentioned LRG sugar-chain alterations. Aberrant glycosylation has been observed in serum glycoproteins of cancer patients. Increased α1–3/4 fucosylation (Lewis X/A) of highly branched N-glycans has been identified in liver and lung cancer [22]. There has also been a report of an association of aberrant glycosylation with promotion of cancer metastasis [36]. LRG–FTG might thus be involved in not only cancer development, but also cancer metastasis.

Sandanayake et al. proposed that the combination of serum LRG, CA19–9, and interleukin-6 was an effective marker, able to distinguish biliary tract cancer from benign biliary disease, which is difficult using CA19–9 alone [37]. This indicates that using multiple markers that combine several characteristic proteins or sugar chains, rather than single markers, could improve the accuracy of diagnosis. The US Food and Drug Association has recently approved a panel of biomarkers to aid in the diagnosis of ovarian cancer prior to surgery [38], the combination of several biomarkers would be used for various cancers. To develop LRG-FTG marker as a clinical test, two approaches can be considered. One is to develop a lectin-antibody sandwich assay, and the other is to simplify LC-MS method. A lectin-antibody sandwich assay is superior in terms of test cost, however, the low specificity of lectins (recognition of a target sugar chain) remains challenging. On the other hand, LC-MS has prevented its clinical use due to its low-throughput, however, ultra-high performance liquid chromatography technique dramatically improved its performance, and its clinical use is now insight.

## Conclusion

Serum LRG–FTG was significantly elevated in CRC patients compared with healthy volunteers, and its accuracy as a CRC tumor marker was equal to that of CEA. Moreover, combined with CEA, it had an excellent profile. LRG–FTG values corresponded well with treatment outcome for the patients with PD. LRG–FTG is expected to be an alternative marker for diagnosis of CRC, however further validation beyond this initial exploratory cohort is warranted.

## Additional files

Additional file 1: **Figure S1**. LRG with biantennary glycans of CRC patients and healthy individuals. (a) A box plot of LRG with biantennary glycans between CRC patients and healthy individuals, (b) The target glycopeptide structure of LRG with biantennary glycans. (PPTX 90 kb)

Additional file 2: Coefficient Optimization using EXCEL Solver. Process of coefficient optimization of combination assay. (XLSX 18 kb)

Additional file 3: **Figure S2**. OPLS-DA analysis using 136 glycopeptides extracted by t-test. (a) A score plot for 80 CRC patients and 50 healthy individuals. (b) R2 and Q2 plot for two components. (C) ROC analysis for OPLS-DA t1 score. (PPTX 140 kb)

Additional file 4: Raw Data. Primary data for analysis. (XLSX 39 kb)

Additional file 5: **Figure S3**. Relationship of serum LRG–FTG and C-reactive protein in 80 CRC patients. Scatter plots showing the relation LRG-FTG and CRP. (PPTX 68 kb)

## Abbreviations

CEA: Carcinoembriyonic antigen
CRC: Colorectal cancer
LRG_FTG: Leucine-rich alpha-2-glycoprotein-1 with fucosylated triantennary N-glycan
