# Supplementary material for: Serum leucine-rich alpha-2-glycoprotein-1 with fucosylated triantennary N-glycan: a novel colorectal cancer marker
Source: BMC Cancer. 2018 Apr 11;18:406. doi: 10.1186/s12885-018-4252-6 (PMC5896117; doi:10.1186/s12885-018-4252-6)
Supplement: Supplementary file 1 — Figure S1. LRG with biantennary glycans of CRC patients and healthy individuals. (a) A box plot of LRG with biantennary glycans between CRC patients and healthy individuals, (b) The target glycopeptide structure of LRG with biantennary glycans. (PPTX 90 kb) [file 12885_2018_4252_MOESM1_ESM.pptx]

## Slide 1
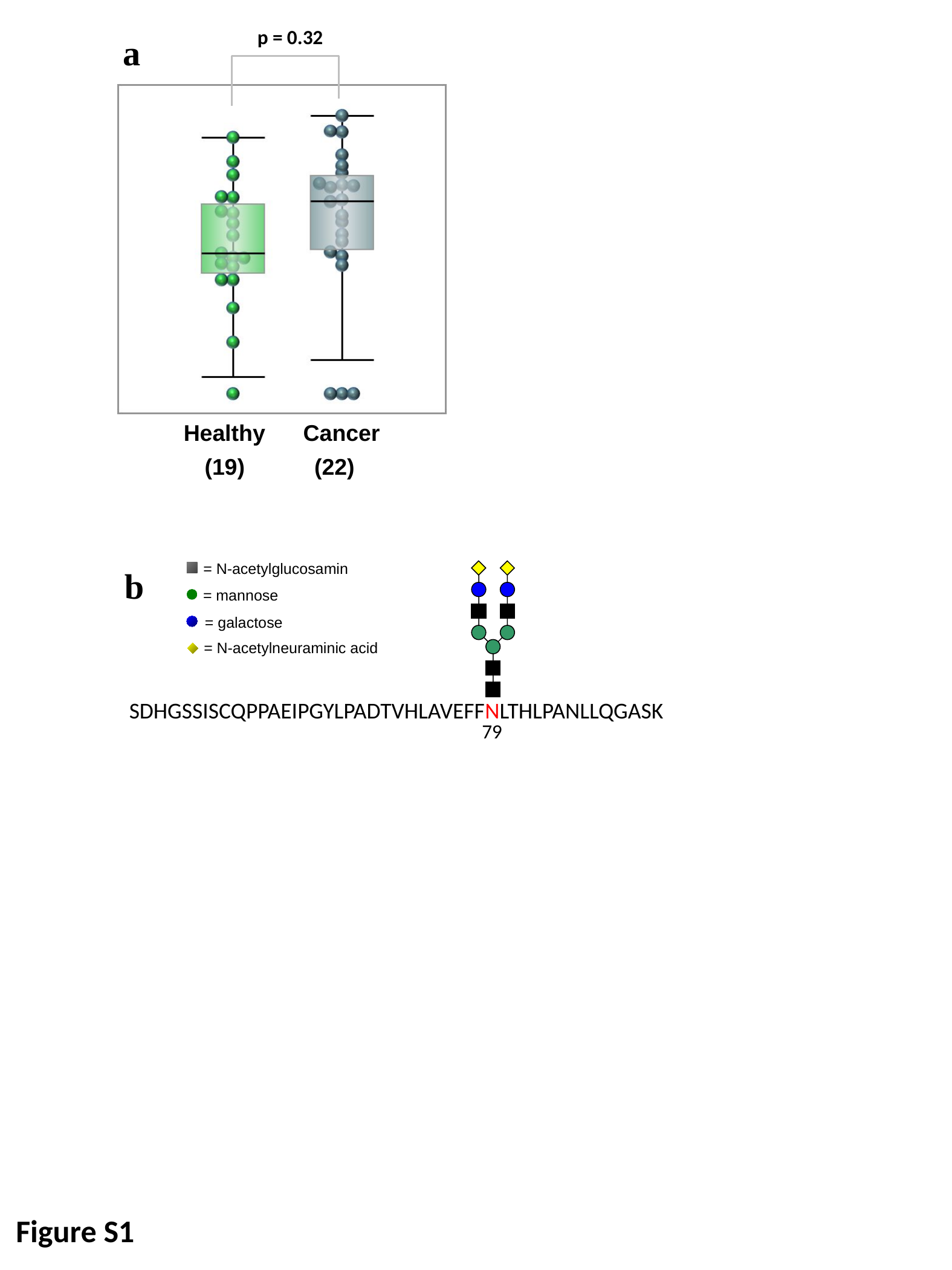

p = 0.32
a
Healthy Cancer
(19) (22)
= N-acetylglucosamin
b
= mannose
= galactose
= N-acetylneuraminic acid
SDHGSSISCQPPAEIPGYLPADTVHLAVEFFNLTHLPANLLQGASK
79
Figure S1
