# Supplementary figures and images for: Serum leucine-rich alpha-2-glycoprotein-1 with fucosylated triantennary N-glycan: a novel colorectal cancer marker
Source: BMC Cancer. 2018 Apr 11;18:406. doi: 10.1186/s12885-018-4252-6 (PMC5896117; doi:10.1186/s12885-018-4252-6)

## Slide 1
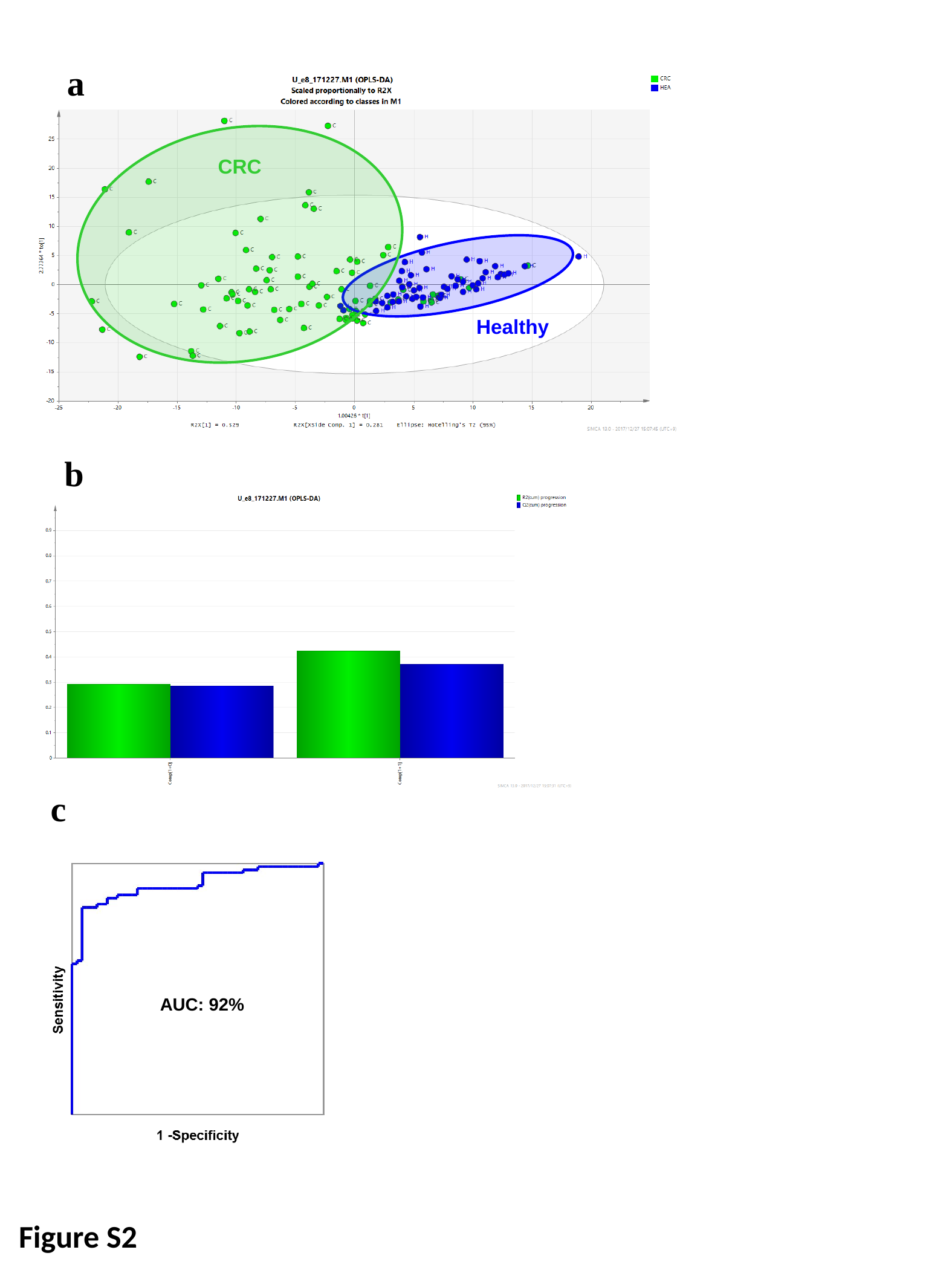

a
CRC
Healthy
b
c
AUC: 92%
Figure S2

Supplement: Supplementary file 3 — Figure S2. OPLS-DA analysis using 136 glycopeptides extracted by t-test. (a) A score plot for 80 CRC patients and 50 healthy individuals. (b) R2 and Q2 plot for two components. (C) ROC analysis for OPLS-DA t1 score. (PPTX 140 kb) [file 12885_2018_4252_MOESM3_ESM.pptx]

## Slide 1
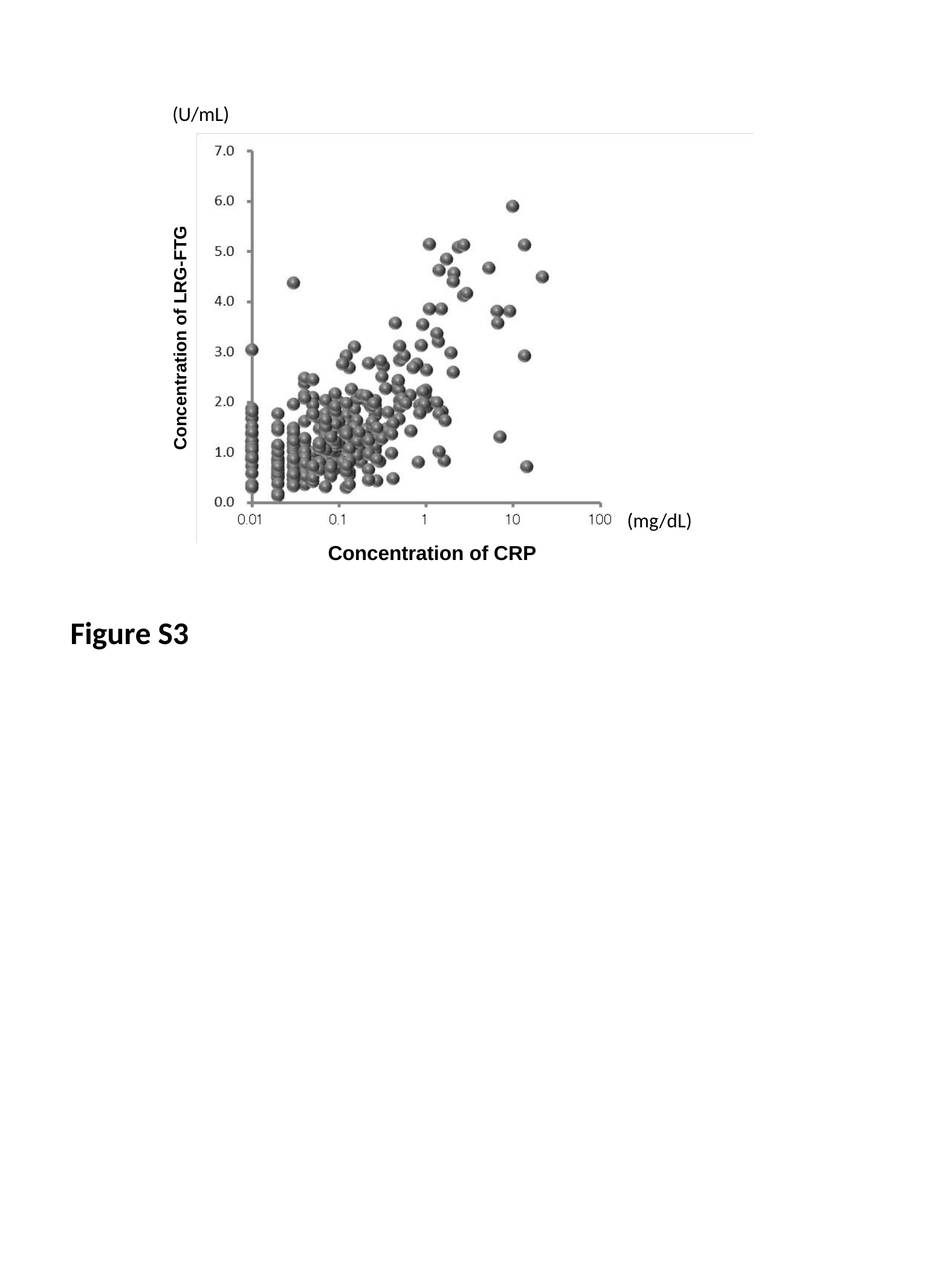

(U/mL)
Concentration of LRG-FTG
(mg/dL)
Concentration of CRP
Figure S3

Supplement: Supplementary file 5 — Figure S3. Relationship of serum LRG–FTG and C-reactive protein in 80 CRC patients. Scatter plots showing the relation LRG-FTG and CRP. (PPTX 68 kb) [file 12885_2018_4252_MOESM5_ESM.pptx]
